# Socioeconomic correlates of incident and fatal opioid overdose among Swedish people with opioid use disorder

**DOI:** 10.1186/s13011-021-00409-3

**Published:** 2021-09-26

**Authors:** Disa Dahlman, Henrik Ohlsson, Alexis C. Edwards, Jan Sundquist, Anders Håkansson, Kristina Sundquist

**Affiliations:** 1 Center for Primary Health Care Research, Department of Clinical Sciences Malmö, Clinical Research Center/CRC, Lund University/Region Skåne, Box 503 22, Malmö, Sweden; 2 Faculty of Medicine, Department of Clinical Sciences Lund, Psychiatry, Lund University, Lund, Sweden; 3 Malmö Addiction Centre, Skåne University Hospital, Malmö, Sweden; 4 Department of Psychiatry, Virginia Institute for Psychiatric and Behavioral Genetics, Virginia Commonwealth University School of Medicine, Richmond, VA USA; 5 Department of Family Medicine and Community Health, Icahn School of Medicine at Mount Sinai, New York, USA; 6 Center for Community-Based Healthcare Research and Education (CoHRE), School of Medicine, Shimane University, Matsue, Japan

**Keywords:** Drug abuse, Opioids, Socioeconomic status, Drug-related death, Poisoning, Sweden

## Abstract

**Background:**

Opioid overdose (OD) and opioid OD death are major health threats to people with opioid use disorder (OUD). Socioeconomic factors are underexplored potential determinants of opioid OD. In this study, we assessed socioeconomic and other factors and their associations with incident and fatal opioid OD, in a cohort consisting of 22,079 individuals with OUD.

**Methods:**

We performed a retrospective, longitudinal study based on Swedish national register data for the period January 2005–December 2017. We used Cox proportional hazard models to investigate the risk of incident and fatal opioid OD as a function of several individual, parental and neighborhood covariates.

**Results:**

Univariate analysis showed that several covariates were associated with incident and fatal opioid OD. In the multivariate analysis, incident opioid OD was associated with educational attainment (Hazard ratio [HR] 0.96; 95% confidence interval [CI] 0.94–0.97), having received social welfare (HR 1.31; 95% CI 1.22–1.39), and criminal conviction (HR 1.53; 95% CI 1.42–1.65). Fatal opioid OD was also associated with criminal conviction (HR 1.93; 95% CI 1.61–2.32).

**Conclusion:**

Individuals with low education and receipt of social welfare had higher risks of incident opioid OD and individuals with criminal conviction were identified as a risk group for both incident and fatal opioid OD. Our findings should raise attention among health prevention policy makers in general, and among decision-makers within the criminal justice system and social services in particular.

**Supplementary Information:**

The online version contains supplementary material available at 10.1186/s13011-021-00409-3.

## Introduction

Drug overdoses (OD) are highly prevalent among people who use drugs; experienced by 17–68% and witnessed by 50–96% [1]. In Sweden, a country with tax-financed healthcare strongly subsidized for the individual [2] and restrictive narcotic policies [3], drug-induced fatalities are the highest in Europe (81deaths/million), and almost four times higher than the European average [4]. In Europe, OD is the single most common cause of death among people with opioid use disorder (OUD), and the mortality among people using opioids is significantly increased compared to the general population [4]. Apart from being potentially fatal, opioid OD can lead to pulmonary, renal and muscular complications as well as anoxia-induced cognitive impairment.

While previous studies have assessed demographic [5–8], psychiatric [9, 10] and substance use related [11–15] risk factors for opioid OD, socioeconomic correlates of OD-related mortality and morbidity have been less thoroughly examined. Associations have been shown between opioid OD and individual-level factors such as criminal justice system involvement [16–19], low income or poverty [8, 20], poor social support e.g. being unmarried, divorced or widowed [5, 8, 21, 22], and low level of educational attainment [5, 8, 20, 21]. On the macro-level, previous research has also shown higher OD rates in deprived areas with high unemployment and low average income [5–7, 23–25], neighborhoods with low educational level [7], and areas with a high percentage of fragmented families [25].

A recent systematic review from 2020 stated that socio-economic marginalization (SEM) is “an important but under-explored determinant of opioid overdose with important implications for health equity and associated public policy initiatives”, and that “There is a critical need for well-designed studies that explicitly and comprehensively examine the association between SEM variables and overdose as their primary purpose” [26].

This is because many previous studies have small sample sizes, compare OD descendants with the general population rather than with people with OUD, or have insufficient adjustment for confounding factors. The knowledge is thus still limited regarding socioeconomic correlates of incident and fatal opioid OD, and studies using nationwide data are highly needed.

In this retrospective nationwide study, we aimed to fill in the knowledge gaps described above by assessing multiple socioeconomic and other factors and their association with incident and fatal opioid OD, in a cohort consisting of 22,079 Swedish individuals with OUD. This is important in order to identify subgroups of people with OUD who could benefit from prioritized opioid OD prevention and take-home naloxone distribution. The opioid antidote naloxone was introduced for take-home use in Sweden in 2018 in order to save people’s lives [27].

## Methods

We collected information on individuals from Swedish population-based registers with national coverage linking each person’s unique personal identification number which, to preserve confidentiality, was replaced with a serial number by Statistics Sweden. We secured ethical approval for this study from the Regional Ethical Review Board of Lund (No. 2008/409, 2012/795, and 2016/679). The database was created by entering all individuals with a registration of opioid use disorder at any time during the study period January 1, 2005 to December 31, 2017. Furthermore, we required that the individual was at least 15 years of age and resided in Sweden at the time of the registration. Opioid use disorder (OUD) was defined as a registration in the Swedish in- and outpatient register by an ICD10 code F11. No exclusion criteria were applied. The study sample thus consisted of both individuals with OUD only, and individuals with OUD in combination with use of other substances.

In the database, we also included the variable opioid overdose (opioid OD). We constructed two different versions of this variable; incident opioid OD (defined in the Swedish National Patient Register for inpatient care and outpatient care, and in conjunction with a non-fatal or fatal outcome), and fatal opioid OD (defined in the Cause of Death register). Incident and fatal opioid OD were defined by the same ICD 10 codes (F11.0, F19.0, X41-X44, Y11-Y14) and incident opioid OD was defined as the first in- or outpatient registration during the study period. Note that if the individual was included in the database by the ICD10 code F11.0 (opioid OD), we required that the next registration of F11.0 occurred at least 7 days afterwards. Our definition of opioid OD included polydrug OD and not only pure opioid OD. This was motivated by toxicology records showing that polydrug fatalities are common and increasing, and that a vast majority (86% in 2018) of fatal poisonings in Sweden involve opioids according to toxicology records [28].

The following individual variables were also added to the database: sex, age at registration of OUD, year of birth, country of birth, criminal conviction, number of years of education, school grades, IQ, resilience, years of education in parent, social welfare, personalized income, neighborhood deprivation, marital status, number of children, distance to mother and distance to father. We also included the following potential confounders: OUD/opioid OD prior to year 2005, inpatient (vs. outpatient) registration of OUD, and psychiatric disorder. For a definition of the variables, see Additional file 1.

We used Cox proportional hazards models to investigate the risk of incident or fatal opioid OD as a function of the covariates described above, from date of OUD registration until end of follow-up (incident or fatal opioid OD, death from other causes, emigration, or December 31, 2017). In the first step we investigated each variable one by one (while controlling for age at registration, year of birth, sex, and prior OUD/opioid OD). The next step was to perform a multivariate stepwise regression model. As several of the variables had a relatively large proportion of missing information, these were not included in the multivariate analysis (education in parent, distance to father/mother, IQ, resilience and school grades). Please note that these variables were not missing due to poor register quality; rather, they were missing because for some of the variables, e.g., IQ, a registration would be needed in the military register and not all individuals were represented in all registers. To summarize the findings with the most parsimonious model possible we used an AIC value model fit to choose the number of parameters to include in the final model [29]. All statistical analyses were performed using SAS software 9.4 [30].

## Results

### Sample characteristics

A total of 22,079 individuals (61.1% male; median age at registration 39.3 ± 16.3 years) with OUD were included in the study (Table 1). A majority were born in Sweden (82.1%), more than half (56.6%) had a criminal registration, 35.3% had received social welfare, 35.4% were married, and 50.6% had children. The median education length was 10.8 ± 2.4 years. Of the entire study sample of people with OUD, 19.6% (*n* = 4320) had at least one registered opioid OD, and 3.4% (*n* = 747) died from opioid OD during the study period.

**Table 1:** Sample characteristics

	All	Data from the Swedish in- and outpatient register				Data from the Swedish Cause of death register			
	*N* = 22,079	No opioid OD *N* = 17,759 (80.4%)		Opioid OD *N* = 4320 (19.6%)		No fatal opioid OD *N* = 21,232 (96.2%)		Fatal opioid OD *N* = 747 (3.4%)	
	Mean/%	N	Mean/%	N	Mean/%	N	Mean/%	N	Mean/%
Male sex	61.1%	17,759	59.2%	4320	68.7%	21,332	60.5%	747	76.9%
Age at OUD registration	39.3 (16.3)	17,759	40.8 (16.8)	4320	33.1 (12.3)	21,332	39.5 (16.4)	747	33.0 (11.2)
Year of birth	1972 (16.7)	17,759	1971 (17.2)	4320	1978 (12.8)	21,332	1972 (16.8)	747	1976 (11.7)
Country of birth									
Sweden	82.1%	14,445	81.3%	3688	85.4%	17,494	82.0%	639	85.5%
Nordic countries	3.4%	629	3.5%	116	2.7%	716	3.4%	29	3.9%
Europe	4.9%	904	5.1%	184	4.3%	1062	5.0%	26	3.5%
Asia	6.9%	1320	7.4%	209	4.8%	1494	7.0%	35	4.7%
Outside Europe/Asia	2.7%	461	2.6%	123	2.9%	566	2.7%	18	2.4%
Criminal conviction	56.6%	17,759	52.6%	4320	73.1%	21,332	55.8%	747	79.1%
Education (years)	10.8 (2.4)	17,061	10.9 (2.4)	4192	10.3 (2.0)	20,513	10.8 (2.4)	740	10.4 (1.9)
School grades	−1.06 (1.1)	7789	− 1.0 (1.1)	2476	− 1.2 (1.1)	9828	−1.1 (1.1)	437	−1.16 (1.1)
IQ	−0.66 (0.9)	4662	−0.62 (0.9)	1314	− 0.79 (0.8)	5675	− 0.65 (0.9)	301	−0.82 (0.8)
Resilience	−0.80 (1.0)	4106	−0.77 (1.0)	1127	− 0.92 (1.0)	4965	−0.79 (1.0)	268	−1.01 (0.9)
Parental education (years)	10.7 (2.5)	14,469	10.6 (2.6)	3891	10.8 (2.4)	17,673	10.7 (2.5)	687	10.7 (2.4)
Social welfare	35.3%	17,397	31.3%	4246	51.5%	20,903	34.7%	740	52.2%
Income	0 (1)	17,397	0.04 (1.1)	4246	−0.15 (0.6)	20.903	0.01 (0.5)	740	−0.16 (0.4)
Neighborhood deprivation	0.81 (2.0)	17,081	0.78 (2.0)	4056	0.96 (2.2)	20,437	0.80 (2.0)	700	1.06 (2.1)
Married	35.4%	17,759	38.7%	4320	22.0%	21,332	35.9%	747	20.6%
1 ≤ children	50.6%	17,759	53.0%	4320	40.6%	21,332	51.1%	747	36.7%
Distance to mother (km)	88.2 (484)	11,428	76 (387)	3225	133 (729)	14,086	88 (482)	567	96 (508)
Distance to father (km)	118.9 (532)	9217	98.7 (444)	2676	156 (761)	11,418	111 (532)	475	115 (552)
Prior OUD/opioid OD	3.0%	17,759	2.4%	4320	5.8%	21,332	20.8	747	41.5%
Inpatient registration of OUD	29.1%	17,759	27.6%	4320	35.0%	21,332	28.8%	747	37.3%
Psychiatric disorder	60.3%	17,759	57.6%	4320	71.1%	21,332	60.1%	747	65.1%

*OD* overdose, *OUD* opioid use disorder

### Correlates of incident opioid OD

In the univariate analysis, incident opioid OD was positively associated with male sex, year of birth, criminal conviction, social welfare, neighborhood deprivation, living 10–50 km or 50+ km from the mother, and living 10–50 km or 50+ km from the father. Incident opioid OD was inversely associated with age at OUD registration, being born in Europe outside the Nordic countries or Asia, educational attainment, school grades, IQ, resilience, parental educational attainment, income, and being married (Table 2). We did not find any significant association between incident opioid OD and having children.

**Table 2:** Associations of incident opioid overdose (OD). *N* = 22,079. Cox Regression Models (Hazard ratios and 95% CIs)

	UNIVARIATE MODELS	MULTIVARIATE MODEL
Male sex	1.43 (1.34; 1.53)*	1.29 (1.20; 1.38)*
Age at OUD registration	0.97 (0.97; 0.97)*	1.04 (1.03; 1.05)*
Year of birth	1.03 (1.03; 1.04)*	1.07 (1.06; 1.08)*
Country of birth		
Nordic countries (vs. Sweden)	1.07 (0.89; 1.30)^a^	1.08 (0.88; 1.32)
Europe (vs. Sweden)	0.75 (0.64; 0.87)^a^*	0.84 (0.72; 0.98)*
Asia (vs. Sweden)	0.58 (0.51; 0.67)^a^*	0.63 (0.55; 0.74)*
Outside Europe/Asia (vs. Sweden)	0.96 (0.80; 1.15)^a^	0.99 (0.82; 1.19)
Criminal conviction	1.85 (1.73; 1.99)^a^*	1.53 (1.42; 1.65)*
Education (years)	0.92 (0.90; 0.93)^a^*	0.96 (0.94; 0.97)*
School Grades	0.91 (0.88; 0.94)^a^*	N/A^b^
IQ	0.87 (0.81; 0.92)^a^*	N/A^b^
Resilience	0.91 (0.86; 0.97)^a^*	N/A^b^
Parental education (years)	0.98 (0.96; 0.99)^a^*	N/A^b^
Social welfare	1.60 (1.51; 1.71)^a^*	1.31 (1.22; 1.39)*
Income	0.75 (0.69; 0.82)^a^*	NS
Neighborhood deprivation	1.02 (1.00; 1.03)^a^*	NS
Married	0.78 (0.72; 0.85)^a^*	NS
1 ≤ children	1.01 (0.94; 1.08)^a^	NS
Distance to mother		N/A^b^
0–10 km (vs. same place)	0.95 (0.88; 1.03)^a^	N/A^b^
10–50 km (vs. same place)	1.18 (1.05; 1.32)^a^*	N/A^b^
50+ km (vs. same place)	1.36 (1.11; 1.66)^a^*	N/A^b^
Distance to father		N/A^b^
0–10 km (vs. same place)	1.06 (0.96; 1.16)^a^	N/A^b^
10–50 km (vs. same place)	1.15 (1.01; 1.30)^a^*	N/A^b^
50+ km (vs. same place)	1.23 (1.00; 1.52)^a^*	N/A^b^
Prior OUD/opioid OD	1.96 (1.73; 2.22)*	1.70 (1.49; 1.94)*
Inpatient registration of OUD	1.31 (1.23; 1.40)^a^*	1.28 (1.20; 1.36)*
Psychiatric disorder	1.96 (1.84; 2.09)^a^*	1.76 (1.64; 1.89)*

*HR* Hazard ratio, *CI* confidence interval, *OD* overdose, *OUD* opioid use disorder, *N/A* not available, *NS* not significant

^a^Controlled for sex, age at registration, year of birth, prior OUD/opioid OD

^b^Variable not included in the multivariable analysis due to a relatively large proportion of missing information. These variables were not missing due to poor register quality; rather, they were missing because not all individuals were represented in all registers (e.g. The Conscript Register includes almost exclusively men)

* *p* < 0.05

In the multivariate analysis, the associations remained significant between incident opioid OD and male sex, age at registration, year of birth, being born in Europe outside the Nordic countries or in Asia, criminal conviction, educational attainment and social welfare.

In addition to the covariates above, multivariate analysis revealed associations between incident opioid OD and prior OUD/opioid OD, inpatient registration of OUD, and psychiatric disorder.

### Correlates of fatal opioid OD

In the univariate analysis, fatal opioid OD was associated with male sex, year of birth, being born in the Nordic countries outside Sweden, criminal conviction and social welfare. Fatal opioid OD was inversely associated with age at OUD registration, being born in Europe outside the Nordic countries or Asia, educational attainment, income and being married (Table 3). We did not find any significant associations with school grades, IQ, resilience, parental educational attainment, neighborhood deprivation, having children, distance to the mother, or distance to the father.

**Table 3:** Associations of fatal opioid overdose (OD). *N* = 22,079. Cox Regression Models (Hazard Ratios and 95% CIs)

	UNIVARIATE MODELS	MULTIVARIATE MODEL
Male sex	2.10 (1.77; 2.49)*	1.77 (1.49; 2.11)*
Age at OUD registration	0.97 (0.97; 0.98)*	0.88 (0.86; 0.90)*
Year of birth	1.02 (1.02; 1.03)*	0.90 (0.88; 0.93)*
Country of birth		
Nordic countries (vs. Sweden)	1.51 (1.04; 2.18)^a^*	NS
Europe (vs. Sweden)	0.59 (0.40; 0.87)^a^*	NS
Asia (vs. Sweden)	0.56 (0.40; 0.79)^a^*	NS
Outside Europe/Asia (vs. Sweden)	0.78 (0.49; 1.26)^a^*	NS
Criminal conviction	2.01 (1.67; 2.41)^a^*	1.93 (1.61; 2.32)*
Education (years)	0.94 (0.91; 0.97)^a^*	NS
School grades	0.95 (0.87; 1.03)^a^*	N/A^b^
IQ	0.89 (0.78; 1.01)^a^	N/A^b^
Resilience	0.88 (0.78; 1.00)^a^	N/A^b^
Parental education (years)	1.00 (0.97; 1.03)^a^	N/A^b^
Social welfare	1.33 (1.15; 1.55)^a^*	NS
Income	0.84 (0.72; 0.97)^a^*	NS
Neighborhood deprivation	1.03 (0.99; 1.06)^a^	NS
Married	0.76 (0.62; 0.94)^a^*	NS
1 ≤ children	0.84 (0.70; 1.00)^a^	NS
Distance to mother		
0–10 km (vs. same place)	0.88 (0.73; 1.06)^a^	N/A^b^
10–50 km (vs. same place)	1.09 (0.83; 1.43)^a^	N/A^b^
50+ km (vs. same place)	1.14 (0.68; 1.92)^a^	N/A^b^
Distance to father		
0–10 km (vs. same place)	0.86 (0.70; 1.06)^a^	N/A^b^
10–50 km (vs. same place)	0.96 (0.72; 1.29)^a^	N/A^b^
50+ km (vs. same place)	1.08 (0.65; 1.79)^a^	N/A^b^
Prior OUD/opioid OD	2.90 (2.28; 3.70)*	2.12 (1.65; 2.72)*
Inpatient registration of OUD	1.23 (1.06; 1.43)^a^*	NS
Psychiatric disorder	1.66 (1.42; 1.93)^a^*	1.59 (1.36; 1.85)*

*HR* Hazard ratio, *CI* confidence interval, *OD* overdose, *OUD* opioid use disorder, *N/A* not available, *NS* not significant

^a^Controlled for sex, age at registration, year of birth, prior OUD/opioid OD

^b^Variable not included in the multivariable analysis due to a relatively large proportion of missing information. These variables were not missing due to poor register quality; rather, they were missing because not all individuals were represented in all registers (e.g. The Conscript Register includes almost exclusively men)

* *p* < 0.05

In the multivariate analysis, fatal opioid OD was associated with male sex, age at registration, and criminal conviction. Fatal opioid OD was also associated with prior OUD/opioid OD and psychiatric disorder in the multivariate analysis.

## Discussion

To the best of our knowledge, this is one of the first nationwide studies to investigate multiple socioeconomic and other factors in relation to incident and fatal opioid OD. We found that criminal conviction, educational attainment and social welfare receipt were associated with incident opioid OD in the multivariate analysis. Criminal conviction was the only variable associated with fatal opioid OD.

While univariate analysis showed that several factors were associated with incident opioid OD (sex, age, country of birth, marital status, income, neighborhood deprivation, distance to mother, distance to father, parental educational attainment, IQ, resilience, school grades) and fatal opioid OD (sex, age, country of birth, marital status, income, educational attainment, and social welfare), statistical significance did not remain in the multivariate analysis for several of these factors. The loss of statistical significance in the multivariate analysis may be because several of the covariates are likely to be correlated (e.g. income and social welfare; education and school grades). However, poor socioeconomic status, operationalized as, e.g., low income or low educational attainment, is associated with a range of negative health outcomes. The current study revealed socioeconomic inequities affecting opioid OD outcomes in a selected, vulnerable subgroup of the population, i.e., people with OUD.

Criminal justice system involvement has been shown to be associated with opioid OD in several studies [16, 17], suggestibly due to decreased opioid tolerance after periods of abstinence during incarceration [31]. International research has shown that the risk of fatal opioid OD is particularly high after periods of abstinence, for example incarceration [18, 19]. In Sweden, however, OD mortality related to prison release does not seem to be as evident as it has been in some other settings. While drug use within prison is poorly examined in Sweden, research has shown that the mean number of days from prison release to OD death is more than 2 years [32]. In our study, criminal justice system involvement was not limited to incarceration. Our positive findings related to criminal justice system involvement might thus reflect both higher risks of opioid OD after abstinence, and an indication of a related marginalization and psychiatric comorbidity including lack of impulse control and high-risk behavior. Future research distinguishing type of crime and penalty would add valuable knowledge to the association found between criminality and opioid OD.

Our finding that educational attainment was inversely associated with incident (in uni- and multivariate analysis) and fatal (in univariate analysis) opioid OD was coherent with previous research [5, 8, 20, 21, 31]. To the best of our knowledge, school grades, IQ, resilience and parents’ educational attainment have not been examined as potential correlates of opioid OD previously. In our study, all these factors were inversely associated with incident but not fatal opioid OD in the univariate analysis.

Individuals who had received social welfare services had higher risk of incident (in uni- and multivariate analysis) and fatal opioid OD (in univariate analysis), while income was associated with incident and fatal opioid OD in the univariate analysis. Associations between individual-level poverty and opioid OD have been shown in several previous studies [20, 31]. The macro-level composite variable neighborhood deprivation was associated with only a slight (HR 1.02) risk increase of incident – but not fatal – opioid OD in this study. This was somewhat different from previous research showing more notable associations between macro-level poverty/low income and opioid OD or associations with fatal opioid OD [5–7, 23–25]. The discrepancy in findings might reflect the societal differences between the study settings. The findings that only social welfare was associated with incident opioid OD in the multivariate analysis might be explained by overlap/correlation of the included covariates. Another potential explanation is that social welfare can be seen as a proxy variable not only for low income, but also for social exclusion and unemployment. Unemployment has been identified as a risk factor for fatal OD in previous research [8, 31]. Social welfare – but not necessarily income – is also likely to covary with homelessness/unstable housing, a factor associated with opioid OD in previous research [17].

Social support and inclusion was in this study operationalized as marital status, parental socioeconomic status and physical distance to parents [33]. Being married was inversely associated with incident and fatal opioid OD in the univariate analysis, which is coherent with several previous studies [5, 8, 21, 22, 31]. Living distant from one’s mother/father was a risk factor for incident but not fatal opioid OD in our study, while no correlations between having children and incident/fatal opioid OD were found. Having children or distance to parents have, to our knowledge, not been subject to opioid OD research previously. None of the covariates concerning social support/inclusion were associated with incident or fatal opioid OD in the multivariate analysis. A potential explanation is that lack of family support does not cause marginalization to the same extent in a welfare state like Sweden, as in societies with less social security. In addition, the study sample in our study consisted exclusively of people with OUD, which on its own might be related to poor social support.

As expected, we found associations between opioid OD and sex, age at OUD registration and year of birth. Male sex has been previously identified as a risk factor for opioid OD [31]. Country of birth was affecting the risk of opioid OD in a somewhat contradictory way. While being born in Asia or Europe outside the Nordic countries was inversely associated with incident and fatal OD, the risk of fatal opioid OD was *higher* in people born in the Nordic countries outside Sweden. Regional drug use patterns and opioid administration routes (e.g. injecting vs. smoking) might explain our findings, but more research is needed.

In addition to our main results, we could reproduce previously identified associations between opioid OD and psychiatric disorder [8–10], prior OD [11, 12] and inpatient registration of OUD [17, 34, 35] in univariate and multivariate analysis. The covariate psychiatric disorder also included substance use disorders apart from OUD, since polydrug use is an established risk factor for fatal opioid OD [13–15].

### Strengths and limitations

The data used in this study was retrieved from national registers of documented high quality. The diagnoses in the Swedish Patient Register for inpatient care have been shown to be valid in 85–95% [36], and the Swedish Total Population Register is nearly 100% complete [37, 38]. Use of nationwide data allow a large sample size. While the large sample size is a strength of our study, it is worth noticing that some of the associations that we found might be of minor clinical importance. For example, resilience and neighborhood deprivation were factors associated with incident opioid OD at hazard ratios with low effect magnitude (HR 0.91 and 1.02, respectively). Even though these associations were statistically significant, they are not likely be of high clinical relevance, but rather an effect of the large sample size. In addition, since our data covered a long time span (2005–2017), the results should be interpreted with some caution. Both our outcome variables (incident and fatal opioid OD) and the exposure variables are dynamic over time, and the level of association between the outcome and exposure variables might thus change during the study period.

Since we limited our study sample to individuals with registered OUD, all incident and fatal opioid ODs in Sweden were not captured. Non-fatal OD is highly prevalent among people who use drugs; experienced by 17–68% and witnessed by 50–96% [1]. In Sweden, self-reports show that over 70% of people injecting heroin have survived at least one OD [39, 40]. These numbers indicate that there may be many OD cases never noted in the patient register. Our findings might thus not be fully translatable to people with OUD but without a registered OUD diagnosis.

We did not discriminate between OD resulting from prescriptions or illicit use. Given the use of register data only, we were not able to control for type of opioid used, or route of administration. This is a limitation to our study, since fatalities due to prescribed opioids are increasing in Sweden [41], and some research has shown differences between the characteristics of fatal illicit vs. prescription OD victims [20]. In addition, we did not adjust for opioid substitution treatment (OST) participation. Retention in OST is associated with reduction of overdose mortality and morbidity [42], but due to limitations of the registers used we did not have access to information regarding OST.

## Conclusions

Our findings have important implications and may help to target prevention and treatment of opioid OD among vulnerable subgroups of people with OUD. More than half of our study sample had a registered criminal conviction, and a third had received social welfare, which indicates that healthcare provided within the criminal justice system and social welfare distribution settings might be efficient arenas for opioid OD prevention. For example, opioid OD prevention including take-home naloxone could be emphasized in criminal justice facilities and provided in social welfare distribution settings.

## Supplementary Information

**Additional file 1.** Definition of the variables used in the study.

## Data Availability

The data used to support the findings of this study are restricted by the Regional Ethical Review Board in Lund, Sweden, in order to protect patient privacy. Data are available from the authors, for researchers who meet the criteria for access to confidential data.

## Abbreviations

CI: Confidence interval
HR: Hazard ratio
OD: Overdose
OST: Opioid substitution treatment
OUD: Opioid use disorder
