# Supplementary material for: Socioeconomic correlates of incident and fatal opioid overdose among Swedish people with opioid use disorder
Source: Subst Abuse Treat Prev Policy. 2021 Sep 26;16:73. doi: 10.1186/s13011-021-00409-3 (PMC8474855; doi:10.1186/s13011-021-00409-3)
Supplement: Supplementary file 1 — Additional file 1. Definition of the variables used in the study. [file 13011_2021_409_MOESM1_ESM.doc]

**Appendix**

**Definition of the variables used in the study**

| **Variable** | **Registers** | **Measured at** | **Definition** | **Missing** |
| --- | --- | --- | --- | --- |
| Opioid use disorder | The Swedish National Patient Register for inpatient care (1964–2016) and outpatient care (2001–2016) | First registration during the years 2005-2017 | ICD10 code F11 (mental and behavioral disorders due to opioid use). | 0% |
| Incident opiod overdose | The Swedish National Patient Register for inpatient care (1964–2016) and outpatient care (2001–2016) | First registration during the years 2005-2017 | ICD10: F11.0 (Mental and behavioral disorders due to use of opioids, acute intoxication); F19.0 (Mental and behavioral disorders due to multiple drug use and use of other psychoactive substances: acute intoxication); X41 (Accidental poisoning by and exposure to antiepileptic, sedative-hypnotic, antiparkinsonism and psychotropic drugs, not elsewhere classified); X42 (Accidental poisoning by and exposure to narcotics and psychodysleptics [hallucinogens], not elsewhere classified); X43 (Accidental poisoning by and exposure to other drugs acting on the autonomic nervous system); X44 (Accidental poisoning by and exposure to other and unspecified drugs, medicaments and biological substances); Y11 (Poisoning by and exposure to antiepileptic, sedative-hypnotic, antiparkinsonism and psychotropic drugs, not elsewhere classified, undetermined intent); Y12 (Poisoning by and exposure to narcotics and psychodysleptics [hallucinogens], not elsewhere classified, undetermined intent); Y13 (Poisoning by and exposure to other drugs acting on the autonomic nervous system, undetermined intent); Y14 (Poisoning by and exposure to other and unspecified drugs, medicaments and biological substances, undetermined intent). | 0% |
| Fatal opioid overdose | The Swedish Cause of Death Register | First registration during the years 2005-2017 | ICD10: F11.0 (Mental and behavioral disorders due to use of opioids, acute intoxication); F19.0 (Mental and behavioral disorders due to multiple drug use and use of other psychoactive substances: acute intoxication); X41 (Accidental poisoning by and exposure to antiepileptic, sedative-hypnotic, antiparkinsonism and psychotropic drugs, not elsewhere classified); X42 (Accidental poisoning by and exposure to narcotics and psychodysleptics [hallucinogens], not elsewhere classified); X43 (Accidental poisoning by and exposure to other drugs acting on the autonomic nervous system); X44 (Accidental poisoning by and exposure to other and unspecified drugs, medicaments and biological substances); Y11 (Poisoning by and exposure to antiepileptic, sedative-hypnotic, antiparkinsonism and psychotropic drugs, not elsewhere classified, undetermined intent); Y12 (Poisoning by and exposure to narcotics and psychodysleptics [hallucinogens], not elsewhere classified, undetermined intent); Y13 (Poisoning by and exposure to other drugs acting on the autonomic nervous system, undetermined intent); Y14 (Poisoning by and exposure to other and unspecified drugs, medicaments and biological substances, undetermined intent). | 0% |
| Country of birth | The Total Population Register |  | The following categories were created: Born in Sweden, Nordic countries, Europe except the Nordic countries, Asia, Outside Europe/Asia. | 0% |
| Criminal conviction | The Crime Register (1973–2011) | Prior to inclusion in the study | Criminal conviction was identified by registration in the Swedish Crime Register, which excluded convictions for minor crimes like traffic infractions. CB is measured using all available criminal conviction types.  The Crime Register covers all convictions in lower court from 1973 to 2011 and the following criminal conviction types were used to define criminal convictions (law and chapter in parentheses): (aggravated) assault (3:5, 3:6); illegal threat (4:5); threats and violence against an officer (17:1, 17:2); intimidation (4:7); [Gross] violation of a person’s/woman’s integrity (4:4a); kidnapping (4:1); illegal confinement or restraint (4:2); (aggravated) robbery (8:5,8:6); illegal coercion (4:4); (aggravated) arson (13:1, 13:2); murder, manslaughter or filicide (3:1, 3:2, 3:3); sexual crimes (excluding prostitution and the buying of sexual services but including child pornography; 6:1–6:10, 6:12, 16:10A); theft of a vehicle (8:1–2, 8:4, 8:7–8); theft (including burglary; 8:1–2,8:4); vandalism (12:1–4); vandalism causing danger to the public, sabotage, hijacking (13:3–10; 5a-b); unlawful entering of a person’s home, trespassing (4:6); fraud (9:1–10); embezzlement (10:1–8; 5a-e); dishonesty/crime towards a creditor (includes forged book-keeping in companies; 11:1–5); and forgery (14:1–10). | 0% |
| Number of years of education | The Total Population Register | Highest education achieved at the end of 2017 | Number of years of education | 4% |
| School grades | School Register | At age 16 | The National School Registry contained educational achievement (a grade point average) for all students at the end of grade nine (usually at age 16). From 1988 to 1997 the score was expressed on a scale between 1 (lowest) and 5 (overall mean was 3.2). From 1998 and onwards the score was expressed on scale between 10 (lowest) and 320 (overall mean was 207). For each year and by gender we standardized the grade score into a Z-score with mean 0 and SD 1. | 56% |
| IQ | The Conscript Register | Conscription (approximately at 19 years of age), in males only | The Swedish military service conscription examination involves a full medical assessment including cognitive function (IQ) measured by four subtests representing logical, spatial, verbal and technical abilities. During the years covered by this study, this examination was required by law; only men of foreign citizenship or those with a severe medical condition or disability were excused. The global IQ score, derived from a summation of the four subtests, was standardized to give a Gaussian distributed score between one and nine. The variable was standardized per year with mean 0 and SD 1. | 74% |
| Resilience | The Conscript Register | Conscription (approximately at 19 years of age), in males only | Resilience is designed to measure the ability to cope with psychologically stressful situations and is assessed on a 1 to 9 graded scale corresponding to a categorized Normal distribution centered at 5. The score is assigned by a psychologist who conducts with every conscript a semi-structured conversation that averages 20–25 minutes. We standardized the variable per year with mean 0 and Std 1. | 77% |
| Years of education in parents | The Total Population Register | Highest education achieved at the end of 2017 | Mean number of years of education for mother and father | 19% |
| Social welfare | Longitudinal integrated database for health insurance and labour market studies (LISA) | Measured the year prior to inclusion in the study | Social assistance is categorized into a binary variable based on whether or not the individual has received any social assistance during a year. Social assistance is defined as financial support under the Social Services Act. You can receive support for your upkeep and for other items that you need to have a reasonable standard of living. Examples of common situations when social assistance is given: As an income supplement to low-income families; for unemployed when other unemployment assistance is not provided or is insufficient; when sickness benefits are insufficient or not provided; to those who are bound by the children in the home and can not get childcare and therefore not can seek work. The variable is recorded at the family level, which means that all individuals in a family with social assistance will, in this report, be counted as recipients of social assistance. | 2% |
| Income | Longitudinal integrated database for health insurance and labour market studies (LISA) |  | Family income was based on the annual family income divided by the number of people in the family (i.e., individual family income per capita). The income parameter also took into consideration the ages of people in the family, using a weighted system whereby small children were given lower weights than adolescents and adults. The calculation procedure was performed as follows: The sum of all family members’ incomes was multiplied by the individual’s age-based consumption weight divided by the family members’ total age-based consumption weight. The variable was standardized per year with mean 0 and SD 1. | 2% |
| Neighborhood deprivation | The Total Population Register, Longitudinal integrated database for health insurance and labour market studies (LISA) | Measured the year prior to inclusion in the study | All individuals reside in a SAMS area. There are approximately 9,200 SAMS throughout Sweden, with an average population of 1,000. These SAMS units were initially created by the Swedish authorities for administrative as well as marketing purposes. We created a neighborhood social deprivation (SD) index for each of the SAMS neighborhoods based on register data for all residents in the neighborhood aged 25-64, i.e., the working-age population, which are assumed to have a stronger impact on the neighborhood than others. The SD composite contained the following derived at baseline: the proportion of residents with low education (9 years or less), the proportion of residents with low household income (below half the median income), the proportion of unemployed residents, and the proportion of individuals on financial assistance. In the model, the composite was kept as a continuous variable, with the SD score ranging between -3 and 11 with higher values indicating greater levels of neighborhood deprivation. | 4% |
| Marital status | The Total Population Register | Measured the year prior to inclusion in the study | Marital status was classified as married, unmarried, divorced or widowed. We used two categories, i.e. married vs unmarried, divorced or widowed. | 0% |
| Number of children | The Total Population Register |  | Number of children at registration for Opioid Use Disorder. Categorized into a binary variable (any children vs no children | 0% |
| Distance to mother | Geographical Register | Measured the year prior to inclusion in the study | Based on coordinates that are available for the entire Swedish population. We divided the variable into 4 groups. Same Place, 0-10 KM, 10-50 KM, 50+ KM | 36% |
| Distance to father | Geographical Register | Measured the year prior to inclusion in the study | Based on coordinates that are available for the entire Swedish population. We divided the variable into 4 groups. Same place, 0-10 KM, 10-50 KM, 50+ KM | 48% |
| Opioid use disorder/Opioid overdose prior to year 2005 | The Swedish National Patient Register for inpatient care (1964–2016) and outpatient care (2001–2016) | Registration sometime between 1997 and 2005 (for the fatal opioid overdose analysis this variable could be measured any time prior to end of follow-up) | ICD10 F11 (mental and behavioral disorders due to opioid use); F19.0 (Mental and behavioral disorders due to multiple drug use and use of other psychoactive substances: acute intoxication); X41 (Accidental poisoning by and exposure to antiepileptic, sedative-hypnotic, antiparkinsonism and psychotropic drugs, not elsewhere classified); X42 (Accidental poisoning by and exposure to narcotics and psychodysleptics [hallucinogens], not elsewhere classified); X43 (Accidental poisoning by and exposure to other drugs acting on the autonomic nervous system); X44 (Accidental poisoning by and exposure to other and unspecified drugs, medicaments and biological substances); Y11 (Poisoning by and exposure to antiepileptic, sedative-hypnotic, antiparkinsonism and psychotropic drugs, not elsewhere classified, undetermined intent); Y12 (Poisoning by and exposure to narcotics and psychodysleptics [hallucinogens], not elsewhere classified, undetermined intent); Y13 (Poisoning by and exposure to other drugs acting on the autonomic nervous system, undetermined intent); Y14 (Poisoning by and exposure to other and unspecified drugs, medicaments and biological substances, undetermined intent). | 0% |
| Inpatient/outpatient registration of the opioid use disorder | The Swedish National Patient Register for inpatient care (1964–2016) and outpatient care (2001–2016) | First registration during the years 2005-2017 | If the registration occurred in the inpatient or outpatient register | 0% |
| Psychiatric disorder | The Swedish National Patient Register for inpatient care (1964–2016) and outpatient care (2001–2016) | Prior to inclusion in the study | Any mental disorder; ICD10 codes: FXX, ICD8, 9: 290-319 | 0% |
